# Integrated Analysis of Carotenoid Metabolism, Lipid Profiles, and Gut Microbiota Reveals Associations Fundamental to Skin Pigmentation in Lingshan Chickens

**DOI:** 10.3390/ani15192832

**Published:** 2025-09-28

**Authors:** Shengting Deng, Weiguang Yang, Shengdi Hu, Long Li, Jianhua He, Guozhi Bian

**Affiliations:** 1 Animal Husbandry and Fisheries Research Center, Guangdong Haid Group Co., Ltd., Guangzhou 511400, China; dengshengting97@163.com (S.D.);; 2 Key Laboratory of Microecological Resources and Utilization in Breeding Industry, Ministry of Agriculture and Rural Affairs, Guangdong Haid Group Co., Ltd., Guangzhou 511400, China; 3 College of Animal Science and Technology, Hunan Agricultural University, Changsha 410128, Chinajianhuahy@hunau.net (J.H.)

**Keywords:** Lingshan chicken, skin color, carotenoids, lipid metabolism, gut microbiota

## Abstract

**Simple Summary:**

Shin color is an important trait affecting the value of Lingshan chickens. By integrating carotenoid metabolism, lipid profiles, and gut microbiota, we found that birds with higher pigmentation had greater carotenoid deposition and distinct microbial compositions. Notably, *Lactobacillus* was positively associated with pigmentation, suggesting that this genus promotes carotenoid absorption and deposition. These findings indicate that *Lactobacillus* may serve as a potential target to improve skin color in Lingshan chickens.

**Abstract:**

Skin color is a crucial phenotypic trait in poultry that influences consumer preference, market value, and breed identification. However, the mechanisms underlying skin color variation in Lingshan chickens remain poorly understood. This study aimed to elucidate the physiological, metabolic, and microbial characteristics associated with skin color differences in male Lingshan chickens. A total of 210 castrated male Lingshan chickens were categorized into white-shanked (WS), yellow-shanked (YS), and red-shanked (RS) groups based on the Roche color fan scores. The results showed that chickens in the YS and RS groups exhibited significantly higher body weights and pigmentation levels in the shank, breast, and abdominal skin compared to those in the WS group (*p* < 0.05). Serum concentrations of triglycerides (TG), total cholesterol (TC), high-density lipoprotein (HDL), low-density lipoprotein (LDL), and very-low-density lipoprotein (VLDL) were markedly elevated in RS chickens. Additionally, carotenoid profiles revealed higher deposition of lutein and β-carotene in the skin and adipose tissues of YS and RS birds. Gene expression analysis indicated differential regulation of carotenoid transport and metabolism-related genes among groups. Furthermore, 16S rRNA sequencing of cecal microbiota revealed significant compositional shifts in microbial communities associated with shank pigmentation. Collectively, these findings suggest that differences in shank color in Lingshan chickens are closely linked to lipid metabolism, carotenoid transport, and gut microbiota composition. This study provides novel insights into the biological mechanisms driving skin pigmentation, offering valuable implications for breeding and functional trait selection in indigenous chicken populations.

## 1. Introduction

Lingshan chicken, a native meat-type breed indigenous to China, is renowned for its distinctive flavor profile and strong environmental adaptability [1]. Compared with commercial broilers, Lingshan chickens have a longer growth cycle, which contributes to the accumulation of more abundant flavor compounds, making them highly favored by consumers. Among the various phenotypic traits of this breed, shank pigmentation is a particularly important external characteristic and serves as a key indicator for distinguishing different strains or individuals [2]. In Lingshan capons, the coloration of the head, beak, shank, claws, breast, abdomen, and cloacal region is widely perceived by consumers as a visual marker of health status and meat quality. Chickens exhibiting golden-yellow pigmentation in these regions are generally more appealing to consumers, especially in southern Chinese provinces such as Guangdong and Guangxi. In addition, chickens with more intensely yellow skin tend to be considered healthier and more acceptable in the market [3]. Given that within-breed variations in myoglobin content are typically minimal, nutritional factors, particularly pigment intake and deposition, are believed to play a dominant role in determining carcass color [4]. In fact, the variation in skin pigmentation among chickens is the result of a complex interplay involving the absorption, transport, metabolism, and deposition of dietary pigments within the body [5,6]. Therefore, investigating the mechanisms of pigment absorption, metabolism, and deposition in Lingshan capons, and elucidating the biological basis underlying the differences in yellow and white skin pigmentation, is of great practical significance. Such research will provide a theoretical foundation for developing optimized pigment additive strategies and nutritional interventions aimed at regulating skin coloration to meet consumer preferences and improve market value.

Skin pigmentation in poultry is a phenotypic trait closely associated with various adaptive and functional roles [7,8]. It is a complex physiological process influenced by multiple factors, including environment, nutrition, and genetics. The yellow or reddish pigmentation of poultry skin and egg yolks primarily originates from the deposition of dietary carotenoids-naturally occurring lipophilic pigments widely found in plants and algae [9]. Common feed ingredients such as corn, alfalfa meal, and marigold are rich in diverse carotenoid compounds [10]. Since poultry cannot synthesize carotenoids endogenously, these pigments must be obtained exogenously through the diet [11]. Once ingested, carotenoids are absorbed in the intestinal tract, transported via the circulatory system, and subsequently deposited in various tissues such as skin, adipose tissue, and egg yolk, imparting characteristic coloration to these sites [12]. The intestinal absorption of carotenoids is mediated by specialized transport proteins, such as scavenger receptor class B type 1 (SCARB1), which facilitates the uptake of lipid micelles containing carotenoids into duodenal epithelial cells [13]. These carotenoids are then incorporated into lipoproteins for systemic distribution to peripheral tissues. Therefore, the bioavailability and transport efficiency of carotenoids are heavily dependent on the abundance and composition of circulating lipoproteins [14]. Moreover, different carotenoid species yield distinct pigmentation effects: lutein and zeaxanthin tend to impart yellow hues, whereas β-cryptoxanthin and β-carotene produce more orange to reddish tones [15]. The overall process of carotenoid utilization involves several tightly regulated steps, including micelle formation, enzymatic digestion, epithelial uptake, intracellular trafficking, and lipoprotein-mediated transport [16]. Disruptions or inefficiencies in any of these stages can significantly affect the extent and intensity of pigment deposition. Despite the recognized role of carotenoids in pigmentation, detailed investigations into the specific types and concentrations of carotenoids present in Lingshan chickens with different shank colors remain limited. Likewise, the metabolic pathways governing carotenoid absorption, transformation, and tissue-specific deposition in these birds are not yet fully understood. These knowledge gaps highlight the need for a more comprehensive analysis of carotenoid distribution and metabolic dynamics in relation to shank pigmentation in Lingshan chickens.

In recent years, the gut microbiota has emerged as a critical factor influencing host physiological metabolism and overall health. It plays indispensable roles in nutrient digestion and absorption, immune modulation, and endocrine regulation [17]. In poultry, the composition and functional activity of the gut microbiota not only affect feed utilization efficiency but may also directly or indirectly regulate carotenoid absorption and metabolism, thereby impacting pigment deposition in the skin [18]. For instance, certain microbial taxa are capable of producing specific enzymes that participate in the degradation, transformation, or solubilization of carotenoids, which can substantially alter their bioavailability [19]. In addition, the gut microbiota has been shown to modulate host lipid metabolism, a process intricately linked to carotenoid transport and tissue deposition [20]. Dysbiosis of the intestinal microbiota may compromise gut barrier integrity and impair nutrient absorption, including the efficient uptake of dietary carotenoids [21]. Given the potential inter-individual variation in gut microbial communities among Lingshan chickens and the far-reaching impact of these microbes on host metabolic function, it is reasonable to hypothesize that the gut microbiota may play a non-negligible role in the fn ormation of different shank pigmentation phenotypes. Therefore, a comprehensive investigation into the composition, diversity, and functional potential of the gut microbiota in Lingshan chickens with distinct shank colors is of great significance for elucidating the microbial mechanisms underlying pigmentation and skin color development.

In summary, although the distinctive shank coloration of Lingshan chickens plays a critical role in determining their market value, the underlying biological mechanisms, particularly the interplay among carotenoid metabolism, lipid transport, and gut microbiota, remain poorly understood. This study aims to systematically elucidate the biological basis of shank pigmentation in Lingshan chickens by integrating these key physiological and metabolic dimensions. Specifically, we will compare growth performance and skin pigmentation characteristics across birds with different shank colors, evaluate differences in serum lipid metabolic profiles, and conduct in-depth analyses of gut microbial composition and its potential influence on pigment deposition. We anticipate that this research will provide novel insights into the physiological and molecular mechanisms governing skin color formation in poultry.

## 2. Materials and Methods

### 2.1. Animal Ethics

The experimental procedures of this study were approved by the Animal Care Committee of Hunan Agricultural University, Changsha, China (Approval Number: 2020-43).

### 2.2. Animals, Experiment Design, Diets, and Management

A total of 210 healthy Lingshan cocks (capons), aged 90 days with similar body weights (1.58 ± 0.17 kg), were selected for this study. All birds had been surgically castrated at 45 days of age. The chickens were randomly assigned to six pens, with 35 birds per pen, at a stocking density of 0.25 m^2^ per bird. The experiment lasted for 60 days, preceded by a 7-day adaptation period. Throughout the experimental period, all birds were fed a basal diet that met or exceeded the nutritional requirements recommended by the Chinese Agricultural Industry Standard for poultry (NY/T 3645-2020) [22] (Table 1).

At the end of the trial, shank skin color of each bird was evaluated using the Roche color fan scoring system. Based on the scores, birds were categorized into three pigmentation groups: white-shanked (WS, scores 1–5; *n* = 30), yellow-shanked (YS, scores 6–10; *n* = 30), and red-shanked (RS, scores 11–15; *n* = 30). From each group, six birds with body weights closest to the group mean were selected for detailed physiological, biochemical, and molecular analyses. This approach was chosen to ensure representativeness and minimize bias from extreme individuals.

All birds were reared on floor litter with ad libitum access to feed and water. The photoperiod was maintained at 18 h of light per day with a light intensity of 10 lx. Environmental conditions were controlled with a temperature range of 20–22 °C and relative humidity of 50–60%. Routine vaccination and disinfection procedures were followed throughout the study.

### 2.3. Sample Collection

At the end of the experiment, Birds were first categorized into WS, YS, and RS groups strictly according to the Roche color fan scoring system. From each group, six birds with body weights closest to the group mean were then selected for detailed analyses, to minimize bias from extreme body weights while ensuring representativeness of the average pigmentation phenotype. Blood was drawn from the wing vein and allowed to clot at room temperature for 2 h, followed by centrifugation at 3500 r/min for 10 min. The resulting serum was aliquoted and stored at −80 °C for further analysis. Subsequently, birds were slaughtered, and samples of the heart, liver, spleen, lungs, kidneys, breast muscle, leg muscle, subcutaneous fat, and abdominal fat were collected. All tissue samples were snap-frozen in liquid nitrogen and then transferred to −80 °C for storage. Additionally, cecal content samples were collected for gut microbiota analysis.

### 2.4. Skin Color

At the end of the experimental period, the skin pigmentation of each bird was assessed using a portable colorimeter (CR-400, Konica Minolta, Tokyo, Japan) to record the CIE L*, a*, and b* values of the shank, subaxillary, breast, and abdominal skin. The instrument was calibrated against a white standard plate prior to each measurement. All measurements were carried out under consistent environmental lighting and by the same trained operator to minimize variability.

Shank skin color was subjectively evaluated using the Roche color fan (DSM Nutritional Products, Basel, Switzerland), a standardized 15-scale visual tool ranging from pale yellow (score 1) to deep orange red (score 15). Each bird’s shank was compared with the fan under identical lighting conditions, and the closest score was assigned by a trained evaluator. Based on these scores, birds were classified into three pigmentation groups: white-shanked (WS; scores 1–5), yellow-shanked (YS; scores 6–10), and red-shanked (RS; scores 11–15).

### 2.5. Serum Parameters

The concentrations of triglycerides (TG), total cholesterol (TC), high-density lipoprotein cholesterol (HDL), and low-density lipoprotein cholesterol (LDL) in serum were determined using enzymatic colorimetric assay kits (Nanjing Jiancheng Bioengineering Institute, Nanjing, China), according to the manufacturer’s instructions. In addition, serum VLDL concentrations were determined using a commercial ELISA kit (Nanjing Jiancheng Bioengineering Institute, Nanjing, China) according to the manufacturer’s instructions.

### 2.6. Pigment Concentrations

Carotenoid concentrations in diet, serum, adipose, and tissue samples were quantified using high-performance liquid chromatography (HPLC, E2695, Waters Corporation, Milford, MA, USA) equipped with a photodiode array detector (PDA). Carotenoids were extracted with ethanol: hexane (1:3, *v*/*v*) containing 0.01% BHT, and the hexane phase was evaporated under nitrogen and re-dissolved in methanol: MTBE (1:1, *v*/*v*). Separation was achieved on a C18 reverse-phase column (SunFire C18, 4.6 × 250 mm, 5 μm, Waters) at 30 °C using a methanol–MTBE–water gradient. Detection wavelengths were set at 450 nm for lutein, zeaxanthin, and β-carotene, and 472 nm for β-cryptoxanthin. Identification was confirmed by retention times and absorption spectra compared with authentic standards.

### 2.7. RT-qPCR Analysis

Total RNA was extracted from the liver, kidney, abdominal fat, and subcutaneous fat tissues, followed by reverse transcription and quantitative real-time PCR (q-PCR), as described by Yang et al. [23]. Briefly, total RNA was isolated using Trizol reagent (Invitrogen, Carlsbad, CA, USA), and RNA concentration and purity were assessed using a NanoDrop 2000 spectrophotometer (Thermo Fisher Scientific, Waltham, MA, USA). Complementary DNA (cDNA) was synthesized using a reverse transcription kit according to the manufacturer’s instructions. Q-PCR was performed using a 2 × SYBR Green PCR Master Mix (Accurate Biotechnology Co., Ltd., Changsha, China).

The thermal cycling conditions were as follows: an initial denaturation at 95 °C for 30 s, followed by 40 cycles of denaturation at 95 °C for 5 s and annealing/extension at 60 °C for 30 s. Primers were designed using Primer Premier 6.0 software (Table 2). The relative mRNA expression levels were calculated using the 2^−ΔΔCt^ method.

### 2.8. Cecal Microbiota

Genomic DNA was extracted using a commercial DNA extraction kit (MP Biomedicals, LLC, Irvine, CA, USA), and its concentration and purity were evaluated using a spectrophotometer. The quality of the extracted genomic DNA was assessed by 1% agarose gel electrophoresis. PCR products were examined using 2% agarose gel electrophoresis, and target bands were excised and purified using the AxyPrep DNA Gel Extraction Kit (Axygen, Union City, CA, USA). Based on preliminary quantification from gel electrophoresis, PCR products were accurately quantified using the QuantiFluor™-ST blue fluorescence quantification system (Promega, Madison, WI, USA). Equimolar pooling of samples was performed according to the required sequencing depth for each sample. Sequencing libraries were then constructed and amplified on the PacBio platform. All sequences were clustered into operational taxonomic units (OTUs) based on different similarity thresholds, with a default cutoff of 98.65% for full-length 16S rRNA gene sequences. Bioinformatic and statistical analyses of OTUs were conducted using QIIME (v1.8.0) and R software (v3.1). Taxonomic annotation of ileal and cecal microbiota was performed using the SILVA 132 rRNA database.

### 2.9. Statistical Analysis

Data were organized using Excel and tested for normality using SPSS 26.0 (IBM Corp., Chicago, IL, USA). One-way analysis of variance (ANOVA) followed by Duncan’s multiple range test was performed to assess differences among treatment groups. Results are presented as mean ± standard error of the mean (SEM), and differences were considered statistically significant at *p* < 0.05. For microbiota and metabolomics data involving multiple comparisons, *p*-values were adjusted using the Benjamini–Hochberg false discovery rate (FDR) method, and results with *q* < 0.05 were considered statistically significant. Spearman’s correlation was used to assess associations between microbial taxa, lipid parameters, and carotenoid concentrations.

## 3. Results

### 3.1. Skin Color Differences in Lingshan Chickens with Different Shank Colors

The skin color differences among Lingshan chickens with different shank colors are presented in Table 3. The results showed that the a* values of the shanks and the b* values of the subaxillary and breast skin in the YS and RS groups were significantly higher than those in the WS group (*p* < 0.05). Roche color fan scores indicated that the YS and RS groups had significantly higher pigmentation scores in the shank, subaxillary, breast, and abdominal skin compared to the WS group (*p* < 0.05).

### 3.2. Effect of Skin Color on Serum Biochemical Parameters in Lingshan Chickens

Differences in serum biochemical parameters among Lingshan chickens with different shank colors are shown in Table 4. Compared with the WS group, the YS and RS groups exhibited significantly higher levels of TC, HDL, and LDL (*p* < 0.05). In addition, serum concentrations of TG, TC, LDL, and VLDL were significantly higher in the RS group than in both the WS and YS groups (*p* < 0.05).

### 3.3. Comparative Analysis of Pigment Deposition in the Serum, Tissues, and Organs of Lingshan Chickens with Different Skin Colors

To further investigate the relationship between pigmentation and metabolic profiles, we quantified the levels of nine pigment-related metabolites in the serum, tissues, and organs of chickens from each group. The results are presented in Table 5 and Table 6. Four carotenoids-lutein, zeaxanthin, β-carotene, and β-cryptoxanthin were detected in the serum. Notably, the concentrations of lutein, zeaxanthin, and β-carotene were significantly higher in the YS group than in the RS and WS groups (*p* < 0.05). In subcutaneous fat, abdominal fat, breast muscle, and leg muscle, only lutein was detected. The RS and YS groups exhibited significantly higher lutein concentrations in subcutaneous fat, abdominal fat, and leg muscle compared to the WS group (*p* < 0.05). In the heart, lutein, α-carotene, and β-carotene were identified. All three carotenoids were significantly more abundant in the RS group than in the YS and WS groups (*p* < 0.05). In the liver, lutein and α-carotene were detected, and their concentrations were significantly elevated in both the RS and YS groups compared to the WS group (*p* < 0.05). In the spleen, lungs, and kidneys, only lutein was detected. The RS group showed significantly higher lutein levels in all three organs than the WS group (*p* < 0.05).

### 3.4. Differential Expression of Pigment Deposition–Related Genes in Lingshan Chickens with Different Skin Colors

**Figure 1:**
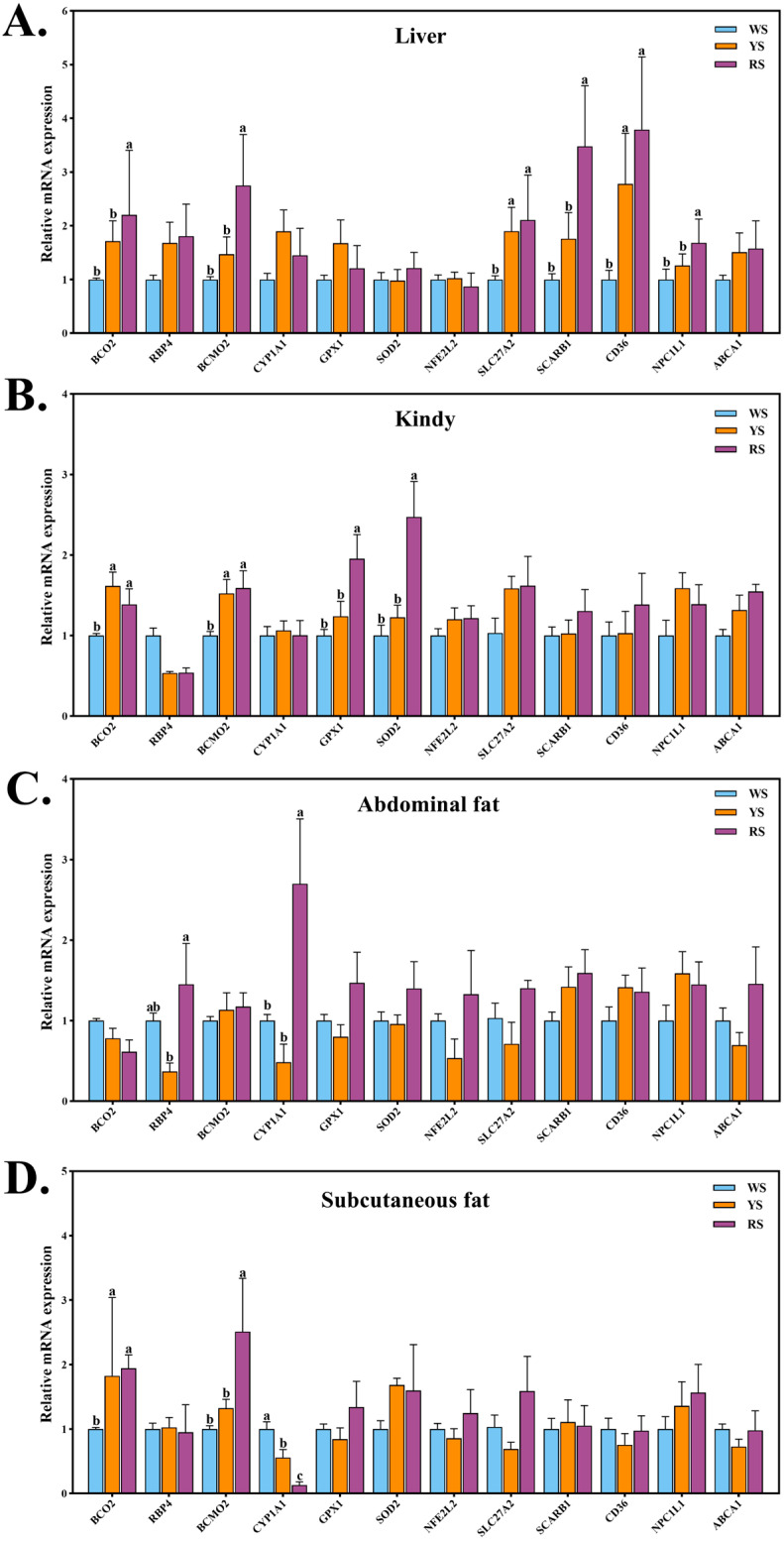
Expression profiles of pigment metabolism-related genes in Lingshan chickens with different skin colors. (**A**) shows the relative mRNA expression of pigment-related genes in the liver. (**B**) presents gene expression patterns in the kidney. (**C**,**D**) display the expression of pigment metabolism–associated genes in abdominal fat and subcutaneous fat, respectively. The values are expressed as mean ± SEM (*n* = 6). SEM, standard error of mean. ^a–c^ Values in the same row with different letter are significantly different (*p* < 0.05). WS, white-shank group; YS, yellow-shank group; RS, red-shank group. BCO2, beta-carotene oxygenase 2; RBP4, retinol-binding protein 4; BCMO1, β-carotene-15,15′-momoxygenase 1; CYP1A1, cytochrome P 450 family 1; GPX1, glutathione peroxidase 1; SOD2, superoxide dismutase 2; NFE2L2, NFE2 like bZIP transcription factor 2; SCARB1, scavenger Receptor Class B Member 1; CD36, CD36 molecule; NPC1L1, Niemann–Pick C1-like 1; ABCA1, ATP binding cassette subfamily A member 1; SLC27A2, solute carrier family 27 member 2.

To further elucidate the mechanisms underlying pigment deposition in Lingshan chickens, we analyzed the mRNA expression of pigment metabolism-related genes in the liver, kidney, abdominal fat, and subcutaneous fat. The results are summarized in Figure 1. In the liver, mRNA expression levels of *BCO2*, *BCMO1*, *SCARB1*, *CD36*, and *NPC1L1* were significantly higher in the RS group compared to the YS and WS groups (*p* < 0.05). In the kidney, *BCMO1* expression was significantly elevated in both the RS and YS groups relative to the WS group (*p* < 0.05). Moreover, *GPX1* and *SOD2* mRNA levels in the RS group were significantly higher than those in the YS group (*p* < 0.05). In abdominal fat, the RS group exhibited significantly higher *CYP1A1* expression than both the WS and YS groups (*p* < 0.05). In subcutaneous fat, *BCMO1* expression was also significantly upregulated in the RS group compared to the other two groups (*p* < 0.05). However, *CYP1A1* expression in subcutaneous fat was significantly lower in the RS and YS groups than in the WS group (*p* < 0.05).

### 3.5. Cecal Microbial Composition in Lingshan Chickens with Different Skin Colors

**Figure 2:**
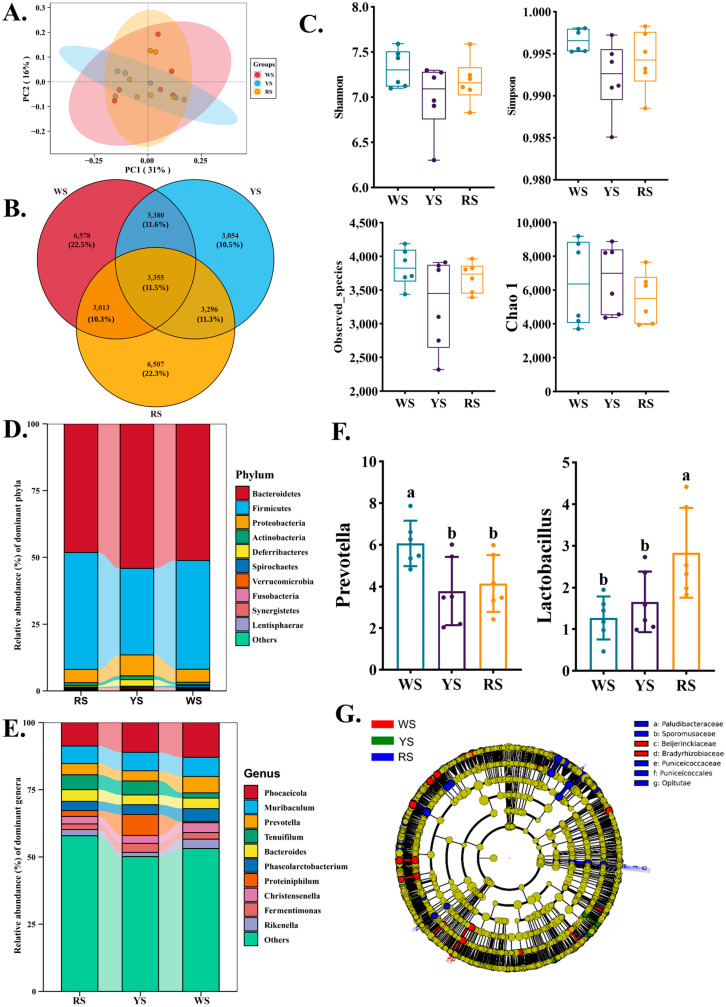
Differences in cecal microbiota composition among Lingshan chickens with different shank colors. (**A**) Principal Coordinates Analysis (PCoA); (**B**) Venn diagram; (**C**) α-diversity analysis of cecal microbiota; (**D**) phylum-level composition of cecal microbiota; (**E**) genus-level composition of cecal microbiota; (**F**) bacteria with significant differences at the phylum level. ^a, b^ Values in the same row with different letter are significantly different (*p* < 0.05); (**G**) LEfSe comparison of gut microbial communities in WS, YS, and RS groups. Panels (a–g) present discriminative taxa across taxonomic levels (features with LDA score > 2.0 and *p* < 0.05 are shown. WS, white-shank group; YS, yellow-shank group; RS, red-shank group.

To investigate whether differences in shank pigmentation were associated with gut microbial composition, we performed 16S rRNA sequencing of cecal contents from chickens in the WS, YS, and RS groups. Principal Coordinates Analysis (PCoA) based on weighted UniFrac distances revealed partial clustering of cecal microbiota among the WS, YS, and RS groups, indicating that shank color may be associated with differences in microbial community structure (Figure 2A). A Venn diagram showed that 3345 operational taxonomic units (OTUs) were shared among all groups, while each group also possessed unique OTUs (Figure 2B). Alpha diversity indices, including Shannon, Simpson, observed species, and Chao1, did not differ significantly among the three groups (*p* > 0.05) (Figure 2C), suggesting that overall microbial richness and diversity were similar. At the phylum level, *Bacteroidetes* and *Firmicutes* were the dominant taxa across all groups, followed by *Proteobacteria* and *Actinobacteria* (Figure 2D) Relative abundance analysis at the genus level revealed that *Phocaeicola* and *Muribaculum* were the most abundant genera, with *Prevotella* being significantly higher in the WS group compared with the YS and RS groups (*p* < 0.05), while *Lactobacillus* was significantly higher in the RS group (*p* < 0.05) (Figure 2E,F). LEfSe analysis further identified discriminative taxa among groups, highlighting family- and genus-level differences associated with shank color (Figure 2G).

### 3.6. Correlation Analysis

**Figure 3:**
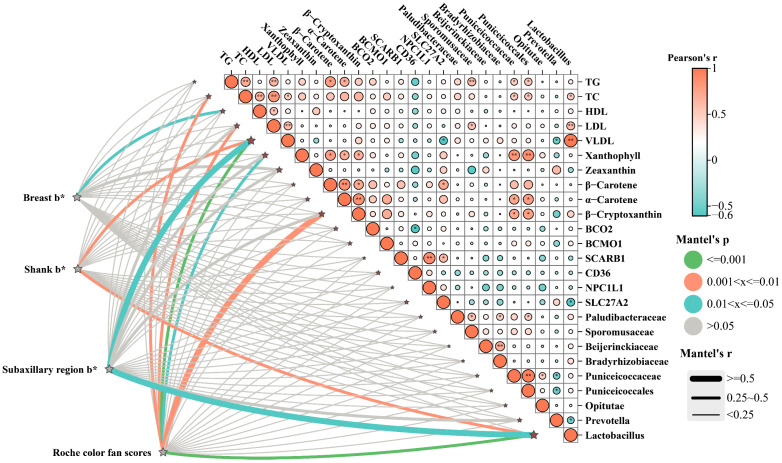
Correlation analysis between skin color and related indicators. * *p* < 0.05, indicates a significant difference; ** *p* < 0.01, indicates a highly significant difference.

Correlation analysis revealed that skin pigmentation traits, represented by Roche color fan scores and b* values of the shank, breast, and subaxillary skin, were strongly associated with carotenoid deposition, lipid profiles, pigment metabolism related genes, and gut microbiota composition (Figure 3). Specifically, Roche color fan scores and shank b* values showed significant positive correlations with xanthophyll and β-cryptoxanthin levels. In terms of gene expression, skin pigmentation was positively associated with *SCARB1*, *CD36*, *NPC1L1*, and *SLC27A2*, which are known to regulate carotenoid uptake and lipid transport, but negatively correlated with *BCO2* expression, an enzyme responsible for carotenoid cleavage. Gut microbiota analysis further revealed that *Lactobacillus* abundances were positively associated with both carotenoid levels and pigmentation scores. Mantel’s test confirmed that variations in gut microbiota were significantly correlated with carotenoid profiles, lipid metabolism, and pigmentation phenotypes.

## 4. Discussion

The observed differences in body weight and skin pigmentation among Lingshan chickens with varying shank colors suggest a potential link between growth performance, carotenoid deposition, and metabolic efficiency. Specifically, birds in the YS and RS groups exhibited significantly greater body weights and higher pigmentation scores than those in the WS group. This finding implies that enhanced nutrient deposition and metabolic activity may contribute to both increased growth and more intense pigmentation phenotypes [24]. The elevated a* and b* values in the shank, subaxillary, and breast skin of YS and RS birds reflect increased deposition of red and yellow pigments, primarily carotenoids. Carotenoids are lipophilic pigments acquired exclusively through the diet, and their deposition in peripheral tissues is closely associated with lipid availability and transport mechanisms [25]. Birds with greater adiposity and lipid metabolism efficiency are more likely to accumulate higher levels of carotenoids in subcutaneous tissues, which may explain the more intense pigmentation observed in YS and RS chickens. Moreover, the higher body weight observed in YS and RS birds may result from a more efficient energy utilization strategy and enhanced nutrient absorption, both of which could facilitate greater deposition of pigments. This is supported by previous studies showing that lipid metabolism is a key determinant of carotenoid transport and tissue distribution in poultry [26]. The positive correlation between body mass and skin pigmentation further underscores the interconnected nature of energy metabolism and pigment deposition in avian physiology [27]. Collectively, these results suggest that differences in shank pigmentation are not solely superficial traits but may reflect underlying variations in growth performance, lipid metabolism, and nutrient utilization efficiency. The integration of phenotypic and metabolic parameters thus offers a more comprehensive understanding of pigmentation mechanisms in native chicken breeds.

The observed alterations in serum biochemical parameters among Lingshan chickens with different shank pigmentation provide further insights into the metabolic basis of pigment deposition. Both the YS and RS groups exhibited significantly elevated levels of TC, HDL, and LDL compared to the WS group, while the RS group showed markedly higher concentrations of TG, TC, LDL, and VLDL relative to both the WS and YS groups. These findings indicate that lipid metabolism plays a central role in the physiological divergence among birds with different pigmentation phenotypes [28]. The increase in circulating lipid fractions, particularly LDL and VLDL, in the RS group suggests enhanced lipid transport capacity, which may facilitate the peripheral distribution of lipophilic compounds such as carotenoids. Previous studies have demonstrated that lipoproteins serve as primary carriers of dietary carotenoids in the bloodstream, enabling their delivery to adipose tissues and integumentary structures where pigmentation manifests [29]. Therefore, the upregulation of serum lipids in more intensely pigmented birds may reflect a greater efficiency in carotenoid mobilization and tissue deposition [27,30]. Moreover, the elevated TG and VLDL levels in the RS group likely indicate increased hepatic lipogenesis and lipid export, processes that are tightly coupled to energy balance and fat accumulation. This metabolic profile is consistent with the observed increase in abdominal fat weight and body mass in RS chickens, suggesting a phenotype characterized by higher anabolic activity and nutrient retention. The positive association between lipid metabolic indices and skin pigmentation supports the hypothesis that pigment accumulation is closely linked to lipid availability and transport efficiency [31]. In summary, the significant upregulation of serum lipid parameters in more pigmented chickens implies a coordinated metabolic adaptation that enhances carotenoid delivery and storage, contributing to the external manifestation of shank pigmentation. These results further reinforce the interconnectedness of lipid metabolism, growth traits, and pigment deposition in poultry.

The differential deposition of carotenoids in the serum, adipose tissues, muscles, and visceral organs among Lingshan chickens with varying shank pigmentation provides critical mechanistic insight into the phenotypic divergence in skin color. As lipid-soluble pigments, carotenoids must be absorbed, transported, and stored in lipid-rich compartments, and their distribution patterns reflect not only dietary intake but also the host’s metabolic efficiency and tissue-specific accumulation capacity [32]. In the present study, four major carotenoids-lutein, zeaxanthin, β-carotene, and β-cryptoxanthin, were detected in the serum, with YS chickens showing significantly higher levels of lutein, zeaxanthin, and β-carotene compared to RS and WS birds. These findings suggest that yellow-shanked chickens possess a more efficient systemic carotenoid absorption or reduced clearance, possibly due to differences in intestinal uptake, lipoprotein binding capacity, or hepatic metabolism. Interestingly, while the RS group exhibited lower serum carotenoid levels than the YS group, it showed higher pigment concentrations in various peripheral tissues, indicating a more efficient carotenoid mobilization and tissue retention mechanism. Among the peripheral depots, adipose tissues (abdominal and subcutaneous fat) and skeletal muscles (breast and thigh) showed exclusive detection of lutein, with significantly higher levels in the YS and RS groups. This is consistent with the lipophilic nature of lutein, which favors accumulation in fatty tissues and muscle membranes [33]. The higher pigment content in RS birds aligns with their elevated serum lipid levels, supporting the notion that lipid abundance enhances carotenoid deposition capacity in target tissues. Notably, the RS group exhibited significantly greater levels of lutein, α-carotene, and β-carotene in the heart and liver, two metabolically active organs involved in nutrient transport, storage, and conversion. The liver, as the central organ for carotenoid metabolism, displayed elevated pigment content in both RS and YS birds, suggesting enhanced hepatic uptake and storage of dietary pigments. The presence of α-carotene and β-carotene-provitamin A carotenoids-exclusively or predominantly in RS chickens may reflect differences in the expression of carotenoid-cleaving enzymes such as BCMO1 and BCO2, which were found to be upregulated in RS livers, as shown in the gene expression analysis [34]. Furthermore, the detection of lutein in the spleen, lungs, and kidneys, organs not classically considered major pigment reservoirs, suggests systemic distribution and possibly antioxidant roles for carotenoids beyond pigmentation [35]. The significantly higher levels of lutein in these organs in the RS group may confer additional physiological advantages, such as enhanced oxidative defense or immunomodulation.

To gain mechanistic insights into the observed differences in carotenoid deposition, we examined the expression of genes involved in carotenoid absorption, transport, cleavage, and oxidative regulation in multiple tissues of Lingshan chickens with varying shank pigmentation. The transcriptional profiles revealed tissue and group-specific expression patterns that mirror the phenotypic differences in pigmentation intensity and pigment distribution. In the liver, the RS group exhibited significantly elevated expression of BCO2, BCMO1, SCARB1, CD36, and NPC1L1, key genes involved in carotenoid metabolism and lipid transport. SCARB1 encodes the scavenger receptor class B type I, which mediates selective uptake of carotenoids and lipids from lipoproteins, while NPC1L1 and CD36 facilitate intestinal and hepatic absorption of cholesterol and carotenoids [36,37]. Their coordinated upregulation suggests enhanced hepatic uptake and mobilization of dietary carotenoids in RS chickens, which may contribute to the observed higher tissue pigment content. Notably, BCMO1 and BCO2, encoding the central and eccentric carotenoid cleavage enzymes, respectively, were also upregulated in the RS group. This suggests that RS chickens not only absorb more carotenoids but may also possess higher capacity to cleave carotenoids into biologically active derivatives such as retinal or apocarotenoids [38]. Such derivatives may exert additional physiological functions, including antioxidant protection and immune modulation, further enhancing the metabolic phenotype of RS birds. Together, these results suggest that the intense pigmentation observed in RS chickens arises from a concerted enhancement of carotenoid uptake, transport, cleavage, and antioxidant defense, particularly in the liver and fat tissues. The fine-tuned expression of pigment-related genes across multiple organs underpins the group-specific differences in carotenoid deposition and provides compelling molecular evidence supporting the phenotypic divergence in skin pigmentation among Lingshan chickens.

The gut microbiota plays a pivotal role in host nutrient metabolism, immune regulation, and even phenotype expression, including pigmentation [20]. In this study, 16S rRNA sequencing revealed that the cecal microbial communities of Lingshan chickens varied significantly among the WS, YS, and RS groups, suggesting a close association between gut microbial composition and shank color phenotypes. Although α-diversity metrics such as Chao1, Shannon, and Simpson indices showed no significant differences among groups, β-diversity analysis via PCA demonstrated clear clustering patterns that separated WS chickens from the more pigmented YS and RS birds. This indicates that while the overall richness and diversity remained comparable, the composition and relative abundance of key taxa differed substantially across pigmentation phenotypes [39]. The distinct microbial profiles observed may underlie differential host metabolic capabilities, particularly in relation to lipid absorption and carotenoid bioavailability. At the phylum level, *Firmicutes* and *Bacteroidetes* dominated the cecal microbiota, as expected [40]. At finer taxonomic resolution, RS birds showed a remarkable enrichment of genera such as *Prevotella sp900547005*, *Sphaerochaeta haiotolerans*, and *Faecalimonas*, taxa known for their involvement in carbohydrate fermentation, SCFA production, and mucosal immunity [41,42]. These functional bacteria may indirectly support pigment deposition by enhancing gut barrier function, increasing antioxidant capacity, or modulating bile acid metabolism, which in turn can affect carotenoid solubilization and absorption. The LEfSe analysis provided robust statistical support for these microbial differences and identified key bacterial taxa with discriminatory power among groups. The enrichment of *Paludibacteraceae*, *Bradyrhizobiaceae*, and *Sporomusaceae* in RS birds further highlights the functional versatility of their microbiota, potentially contributing to the higher efficiency of nutrient utilization and pigment deposition observed in this group. In addition, *Lactobacillus* abundance was positively correlated with carotenoid deposition and pigmentation scores, suggesting that this genus may facilitate intestinal carotenoid absorption and deposition in peripheral tissues. One possible explanation is that *Lactobacillus* may influence bile acid metabolism, which in turn could modulate carotenoid solubilization and absorption, as suggested by previous studies in poultry and mammals. However, since bile acid profiles and bile salt hydrolase activity were not assessed in the present study, this proposed pathway remains speculative and warrants further investigation. In summary, the gut microbial landscape in Lingshan chickens appears to be closely linked with shank pigmentation phenotypes. These findings should be interpreted as associations, and further intervention studies will be required to confirm causality. The distinct microbial consortia observed in RS, YS, and WS birds likely contribute to the differential metabolic environments that facilitate or limit carotenoid uptake, transformation, and deposition. These findings provide new perspectives on the role of the gut microbiota in avian phenotypic expression and underscore its potential as a target for nutritional or microbial modulation strategies to enhance desirable traits such as skin pigmentation.

## 5. Conclusions

This study comprehensively elucidates the physiological, metabolic, and microbial mechanisms underlying skin color variation in Lingshan chickens. Birds with red and yellow shanks exhibited greater body weights, increased serum lipid levels, and higher tissue deposition of carotenoids, particularly lutein and β-carotene. These phenotypes were associated with elevated expression of key carotenoid transport and metabolism-related genes in the liver and adipose tissues. In parallel, distinct cecal microbiota profiles were observed across pigmentation groups, with RS birds harboring microbial communities enriched in taxa related to lipid metabolism and nutrient assimilation. Correlation analysis confirmed the involvement of *Lactobacillus* in promoting pigment deposition. These findings demonstrate that skin pigmentation in Lingshan chickens is regulated by a complex interplay of lipid metabolic pathways, pigment transport gene expression, and gut microbial composition. In addition, future studies should validate these mechanisms across breeds and test nutritional or probiotic strategies to enhance pigmentation.

## Figures and Tables

**Table 1:** Basal diet composition (air-dry basis).

Items	Content
Ingredients (%)	
Corn	53.85
Sorghum	20.00
Soybean meal	16.50
Fish meal	4.20
Calcium hydrogen phosphate	1.00
Stone powder	0.90
Dl-methionine	0.15
L-Lysine sulfate	0.50
L-Threonine	0.08
Salt	0.20
Choline chloride	0.10
Complex enzyme	0.02
Premix ^1^	2.50
Total	100.00
Nutrition level ^2^	
Metabolizable energy, (MJ/Kg)	12.76
Crude protein, %	16.20
Calcium, %	1.35
Total phosphorus, %	0.42
Available phosphorus, %	0.33

^1^ The premix provided the following per kilogram of diet: Vitamin A, 16,500 IU; Vitamin D_3_, 1900 IU; Vitamin E, 18 IU; Vitamin K_3_, 4.0 mg; Vitamin B_1_, 1.7 mg; Vitamin B_2_, 9.0 mg; Vitamin B_6_, 20.02 mg; Vitamin B_12_, 12 μg; D-calcium pantothenate, 40 mg; Folic acid, 1.6 mg; Niacin, 55 mg; Biotin, 0.22 mg; Cu (as copper sulfate), 112.5 mg; Zn (as zinc oxide), 110 mg; Mn (as manganese sulfate), 63.5 mg; I (as calcium iodate), 0.66 mg; Se (as sodium selenite), 0.4 mg; Canthaxanthin (2.5%), 0.1 g; Natural lutein (2.0%), 0.2 g. ^2^ Nutritional levels, except for crude protein, calcium, and total phosphorus which are measured values, are all calculated values.

**Table 2:** Nucleotide sequences of primers used to measure targeted genes.

Gene Symbols	Accession NO.	Production Length	Primer Sequence (5′ to 3′)
*SCARB1*	XM_015275627.4	141	GTGAACCAGCGTGGACCGTATG CTCATCTTCAGTGCCGTTGGACAA
*CD36*	NM_001030731.1	132	GCGATTTGGTTAATGGCACTGATGG CCTTCACGGTCTTACTGGTCTGGTA
*NPC1L1*	NM_207242.2	105	CTGGCTGGCTCTCATCATCATCTTC CCTGCTGTCTTGTTCTTGTTCCTGT
*ABCA1*	NM_204145.3	225	GCTCTCCGAAGTGGCTCTGATGA ACGAGTGTGGCTGGAACGATGTA
*BCO2*	XM_004948142.5	136	GATTAACAACCAGCACAACCGCATT GCTCACATTGGCATTGTCACTTGG
*RBP4*	NM_205238.2	55	ACTGGGTAGTGGACACAGATTACGA TCACGGCAGGAATAATGAAGAGCAT
*SLC27A2*	XM_046934089.1	104	ACCAACACTACCACTGCCTCCAA GCCACCATCATCACCTTCATCTCTG
*BCMO1*	NM_001364902.2	149	CAAAGAAGAGCATCCAGAGCCCATA GCAGCAGAGCCAAGCCATCAA
*CYP27B1*	NM_010009.2	403	TCCAGAGGCAGTGAGTCGGTTC CGTTGTCCAGAGTTCCAGCATAGC
*CYP1A1*	NM_205147.2	116	TCTTCCTCTTCCTCACCACCATCC GCACTCGCACTGCTTGTACTTCA
*GPX1*	NM_001277853.3	166	GCAAAGTGCTGCTGGTGGTCAA ATCTCCTCGTTGGTGGCGTTCT
*SOD2*	NM_204211.2	188	GTCGCAAGGCAGAAGCACACT GACACCTGAGCTGTAACATCACCTT
*NFE2L2*	NM_001396902.1	93	GGACGGTGACACAGGAACAACAA CTCCACAGCGGGAAATCAGAAAGAT
*β-actin*	NM_205518.2	89	CCAGCCATGTATGTAGCCATCCAG ACACCATCACCAGAGTCCATCACA

SCARB1, scavenger Receptor Class B Member 1; CD36, CD36 molecule; NPC1L1, Niemann–Pick C1-like 1; ABCA1, ATP binding cassette subfamily A member 1; BCO2, beta-carotene oxygenase 2; RBP4, retinol-binding protein 4; SLC27A2, solute carrier family 27 member 2; BCMO1, β-carotene-15,15′-momoxygenase 1; CYP27B1, cytochrome P450 family 27 subfamily B member 1; CYP1A1, cytochrome P 450 family 1; GPX1, glutathione peroxidase 1; SOD2, superoxide dismutase 2; NFE2L2, NFE2 like bZIP transcription factor 2.

**Table 3:** Skin Color Differences Among Lingshan Chickens with Different Shank Colors.

Items ^1^	WS	YS	RS	SEM ^2^	*p*-Value
a*					
Shank	10.14 ^b^	12.53 ^b^	16.78 ^a^	0.97	0.009
Subaxillary region	3.83	4.29	3.39	0.296	0.506
Breast	8.83	7.14	9.76	0.699	0.319
b*					
Shank	29.70	31.74	32.74	0.921	0.413
Subaxillary region	4.14 ^b^	10.68 ^a^	11.29 ^a^	1.068	0.009
Breast	15.85 ^b^	20.88 ^a^	22.12 ^a^	0.994	0.006
L*					
Shank	66.20	68.66	61.83	1.311	0.105
Subaxillary region	71.12	72.23	73.94	1.054	0.574
Breast	65.94	70.81	67.92	0.922	0.088
***Roche color fan scores***					
Shank	4.17 ^c^	8.67 ^b^	11.67 ^a^	0.768	<0.001
Thigh	1.00	1.17	1.33	0.090	0.342
Subaxillary region	1.00 ^b^	1.67 ^a^	2.00 ^a^	0.145	0.007
Breast	1.33 ^b^	3.17 ^a^	3.83 ^a^	0.298	<0.001
Abdomen	2.33 ^b^	4.17 ^a^	4.00 ^a^	0.283	0.005
Back	1.00	1.33	1.17	0.090	0.342

^1^ Results are presented as mean ± standard error (*n* = 6). ^2^ SEM, standard error of mean. ^a–c^ Values in the same row with different letters are significantly different (*p* < 0.05). WS, white-shank group; YS, yellow-shank group; RS, red-shank group.

**Table 4:** Differences in Serum Biochemical Parameters Among Lingshan Chickens with Different Skin Colors.

Items ^1^	WS	YS	RS	SEM ^2^	*p*-Value
TG (mmol/L)	0.47 ^b^	0.65 ^b^	1.03 ^a^	0.075	0.002
TC (mmol/L)	1.83 ^c^	2.66 ^b^	3.65 ^a^	0.194	<0.001
HDL (mmol/L)	3.81 ^b^	4.72 ^a^	5.07 ^a^	0.193	0.006
LDL (mmol/L)	1.51 ^b^	2.39 ^a^	2.67 ^a^	0.150	<0.001
VLDL (mmol/mL)	0.54 ^b^	0.55 ^b^	0.57 ^a^	0.057	0.008

^1^ Results are presented as mean ± standard error (*n* = 6). ^2^ SEM, standard error of mean. ^a–c^ Values in the same row with different letters are significantly different (*p* < 0.05). WS, white-shank group; YS, yellow-shank group; RS, red-shank group. TG, triglyceride; TC, Total cholesterol; HDL, high-density lipoprotein; LDL, low-density lipoprotein; VLDL, very low-density lipoprotein.

**Table 5:** Differences in Pigment Content in the Serum and Tissues of Lingshan Chickens with Different Skin Colors.

Items ^1^	WS	YS	RS	SEM ^2^	*p*-Value
Xanthophyll, µg/100 mg					
Serum	0.0030 ^c^	0.0125 ^b^	0.0202 ^a^	0.0028	0.0040
Subcutaneous fat	0.0022 ^b^	0.0049 ^a^	0.0052 ^a^	0.0005	0.0060
Abdominal fat	0.0022 ^b^	0.0069 ^a^	0.0062 ^a^	0.0008	<0.001
Breast muscle	0.0000 ^b^	0.0010 ^a^	0.0013 ^a^	0.0002	0.0140
Thigh muscle	0.0015	0.0038	0.0021	0.0008	0.5690
Zeaxanthin, µg/100 mg					
Serum	0.0021 ^c^	0.0071 ^b^	0.0135 ^a^	0.0018	0.0060
Subcutaneous fat	0.0000	0.0000	0.0000	-	-
Abdominal fat	0.0000	0.0000	0.0000	-	-
Breast muscle	0.0000	0.0000	0.0000	-	-
Thigh muscle	0.0000	0.0000	0.0000	-	-
β-Cryptoxanthin, µg/100 mg					
Serum	0.0000	0.0010	0.0009	0.0002	0.0920
Subcutaneous fat	0.0000	0.0000	0.0000	-	-
Abdominal fat	0.0000	0.0000	0.0000	-	-
Breast muscle	0.0000	0.0000	0.0000	-	-
Thigh muscle	0.0000	0.0000	0.0000	-	-
β-Carotene, µg/100 mg					
Serum	0.0000 ^b^	0.0002 ^b^	0.0013 ^a^	0.0002	0.0030
Subcutaneous fat	0.0000	0.0000	0.0000	-	-
Abdominal fat	0.0000	0.0000	0.0000	-	-
Breast muscle	0.0000	0.0000	0.0000	-	-
Thigh muscle	0.0000	0.0000	0.0000	-	-

^1^ Results are presented as mean ± standard error (*n* = 6). ^2^ SEM, standard error of mean. ^a–c^ Values in the same row with different letters are significantly different (*p* < 0.05). WS, white-shank group; YS, yellow-shank group; RS, red-shank group.

**Table 6:** Differences in Pigment Content in the Organs of Lingshan Chickens with Different Skin Colors.

Items ^1^	WS	YS	RS	SEM ^2^	*p*-Value
Xanthophyll, µg/100 mg					
Heart	0.0030 ^c^	0.0125 ^b^	0.0202 ^a^	0.0027	0.0044
Liver	0.0090 ^b^	0.0167 ^ab^	0.0308 ^a^	0.0039	0.0398
Spleen	0.0026 ^b^	0.0116 ^b^	0.0293 ^a^	0.0046	0.0213
Lung	0.0009 ^b^	0.0022 ^b^	0.0045 ^a^	0.0006	0.0021
Kidney	0.0000 ^b^	0.0029 ^ab^	0.0037 ^a^	0.0008	0.0487
α-Carotene, µg/100 mg					
Heart	0.0000 ^b^	0.0000 ^b^	0.0038 ^a^	0.0007	0.0046
Liver	0.0000 ^b^	0.0011 ^ab^	0.0016 ^a^	0.0003	0.0460
Spleen	0.0000	0.0000	0.0000	-	-
Lung	0.0000	0.0000	0.0000	-	-
Kidney	0.0000	0.0000	0.0000	-	-
β-Cryptoxanthin, µg/100 mg					
Heart	0.0005 ^b^	0.0029 ^b^	0.0067 ^a^	0.0010	0.0081
Liver	0.0000	0.0000	0.0000	-	-
Spleen	0.0000	0.0000	0.0000	-	-
Lung	0.0000	0.0000	0.0000	-	-
Kidney	0.0000	0.0000	0.0000	-	-

^1^ Results are presented as mean ± standard error (*n* = 6). ^2^ SEM, standard error of mean. ^a–c^ Values in the same row with different letters are significantly different (*p* < 0.05). WS, white-shank group; YS, yellow-shank group; RS, red-shank group.

## Data Availability

The data presented in this study were available on request from the corresponding author.
